# Negatively charged phospholipids doped liposome delivery system for mRNA with high transfection efficiency and low cytotoxicity

**DOI:** 10.1080/10717544.2023.2219869

**Published:** 2023-06-12

**Authors:** Lin Wang, Huanchun Xing, Shuai Guo, Wenbin Cao, Zinan Zhang, Lijuan Huang, Sui Xin, Yuan Luo, Yongan Wang, Jun Yang

**Affiliations:** a State Key Laboratory of Toxicology and Medical Countermeasures, Institutes of Pharmacology and Toxicology, Academy of Military Medical Sciences, Beijing, China; b Tianjin University of Science and Technology, Tianjin, China; c Hebei University of Science and Technology, Shijiazhuang, China

**Keywords:** Liposome, mRNA transfection, nanocarrier, lipid nanoparticle, mRNA drug

## Abstract

Messenger RNA (mRNA) has become one of the most potential drugs in recent years. However, efficient and safe delivery of fragile and easily degradable mRNA is a major challenge. Appropriate delivery system (DS) determines the final effect of mRNA. Cationic lipids play a crucial and decisive role in the entire DS, but also cause huge biosafety problems due to the high toxicity. In this study, a new DS for mRNA delivery that combines negatively charged phospholipids was developed in order to neutralize the positive charge and thus increase the safety. Further, the factors affecting mRNA transfection from cell to animal were investigated. The mRNA DS with optimum condition of lipid composition, proportions, structure, and transfection time was synthesized. Adding an appropriate amount of the anionic lipid to liposomes could increase the safety while maintaining the original transfection efficiency. For transporting mRNA in vivo, requirements regarding the mRNA encapsulation and releasing rate should be further considered to optimize DS design and preparation.

## 1. Introduction

Messenger RNA (mRNA) can be used to treat diseases by allowing cellular translation of the proteins they encode, and they are currently some of the most promising drugs in development (To & Cho, 2021; Webb et al., 2022). Translation of mRNA occurs in the cytoplasm without entering the nucleus compared with DNA drugs which have the risk of integration into the genome, leading to much safer (Schlake et al., 2012; Anthony, 2022). Unfortunately, naked mRNA is extremely fragile and easily degraded by a variety of biological enzymes because of its long, single-stranded structure (Eygeris et al., 2022). Moreover, it is extremely difficult for negatively charged mRNAs to penetrate the cell membrane (Koynova & Tenchov, 2011; Durymanov & Reineke, 2018). Even in cells, mRNA needs to be effective against lysosomal destruction (Forster Iii et al., 2022). Therefore, an effective mRNA delivery system is essential to maintaining stable mRNA structure and ensuring effective transfection (Ramachandran et al., 2022).

Nanocarriers, such as micelles, nanoemulsion, cationic polymers, protamine, and lipids, have been widely developed for mRNA delivery (Li et al., 2021, 2022a). Among these, liposomes are currently the most mature and widely used, and they have the best comprehensive performance and large-scale mass production (Tenchov et al., 2021). Generally, cationic lipids are used as the core component and play a crucial and decisive role in the entire delivery system owing to their encapsulation of negative mRNA, penetration across the cell membrane, and lysosomal escape via the proton sponge effect (Bogaert et al., 2022). However, while cationic lipids have high efficacy, they also cause huge biosafety problems owing to their high toxicity (Zhang et al., 2004; Lv et al., 2006). Currently, the application of ionizable lipids to construct liposomes is a relatively successful and mature solution that has been applied practically in the production of mRNA vaccines (Ramachandran et al., 2022). However, the liposome system has extremely high requirements for details of composition, proportion, mRNA loading mode, synthesis method, solution environment, purification method, among other considerations, which have a crucial impact on the final mRNA transfection (Carrasco et al., 2021). Overall, delivery system design and reduction of the high toxicity of the delivery system caused by positively charged lipids while ensuring effectiveness is a key and urgent problem for the development of mRNA drugs (Seki, 2018).

Herein, we developed a new method for mRNA delivery that combines negatively charged phospholipids with traditional cationic lipids in order to neutralize the positive charge and thus increase the safety of the liposome delivery system (Leventis & Grinstein, 2010). Furthermore, we investigated the factors affecting mRNA transfection in vitro and attempted to distinguish the differences between liposome transfection in vitro and in vivo.

## 2. Materials and methods

### 2.1. Cell culture

Neuro-2a cells were cultured in 96-well plates at a density of 3 × 10^5^ cells/mL in Minimum Essential Media (MEM) containing 10% fetal bovine serum (FBS) and 1% Penicillin-Streptomycin for mRNA transfection.

### 2.2. Preparation of liposomes loading EGFP mRNA

**DOTAP/POPS liposomes with different molar ratios and weight:** DOTAP and POPS were weighed and mixed at molar ratios of 9:1, 8:2, 7:3, 6:4, 5:5, 4:6, 3:7, 2:8, 1:9 (8.02:1, 3.56:1, 2.079:1, 1.34:1, 0.89:1, 0.59:1, 0.38:1, 0.22:1, 0.099:1 w/w, respectively), dissolved in chloroform and then remove the organic solvent to form a uniform film by rotary evaporation. In order to investigate the effect of different molar ratios, the concentration of total liposomes (including DOTAP and POPS) remained at 0.48 mg/mL. The Enhanced green fluorescent protein (EGFP) mRNA was dissolved in RNase-free water at the concentration of 0.027 mg/mL. The mRNA solution was mixed with the lipid film and prepared into liposomes by ultrasound in ice bath for half an hour. For further investigating the optimal lipid concentrations at the fixed DOTAP and POPS molar ratio (7:3), the total concentrations of all liposomes were controlled at 0.672, 0.576, 0.48, 0.384, 0.288, 0.192, and 0.096 mg/mL by adjusting the weight of the total lipids when mixing with mRNA solution of the same concentration and volume.

Cholesterol was further added into liposome based on the fixed DOTAP and POPS molar ratio (7:3). DOTAP, POPS, and cholesterol were weighted and mixed at molar ratios of 7:3:0, 7:3:1, 7:3:3, 7:3:7, 7:3:14, and 7:3:21 and prepared into liposome as described above. In order to investigate the effect of different molar ratios, the concentration of total liposomes (including DOTAP, POPS and cholesterol) remained at 0.48 mg/mL. For further investigating the optimal lipid concentrations at the fixed DOTAP, POPS and Cholesterol molar ratio (7:3:14), the total concentrations of all liposomes were controlled at 0.72, 0.48, 0.384, 0.192, 0.096, 0.048, and 0.024 mg/mL also by adjusting the weight of the total lipids when mixing with mRNA solution of the same concentration and volume.

**DOTAP/POPS liposomes with different net charges and structure:** for investigating the net charges of the liposome, DOTAP and POPS were weighed and mixed at molar ratios of 7:0, 7:3, 7:7, and 7:14, as same as before. But the mass of cationic lipid DOTAP keep the same at 0.1328 mg/mL, while the mass of POPS was 0, 0.064, 0.149, and 0.3 mg/mL, respectively. The lipids were prepared into liposomes as described above.

To explore the influence of liposome structure, a series of complexes of liposome and mRNA were prepared. The blank liposomes at the DOTAP and POPS molar ratio (7:3, 10:0, 0:10, 3 groups) were prepared as the same as before, only the pure RNase-free water without EGFP mRNA was applied. And then the EGFP mRNA solution was added after the blank liposome formed, to be adsorbed on the surface of the particles. Furthermore, a mixed liposomes system with blank DOTAP liposomes and POPS liposomes at the molar ratio 7:3 also prepared to adsorb the mRNA on the surface of the liposomes. The final concentration of the liposomes and mRNA were kept at 0.192 and 0.027 mg/mL, respectively.

### 2.3. Cell uptake, transfection and imaging

The liposomes were prepared by ultrasonic method in the conditions of DOTAP and POPS molar ratio (7:3), total lipid concentration (0.192 mg/mL) and EGFP mRNA concentration (0.027 mg/mL), and then were added to cultured cells in 96-well plates at a volume of 150 μL per well after replacing the new MEM for 0.5, 1, 2, or 4 h. Subsequently, all fluids in each group of wells were removed. The cells were washed twice with phosphate-buffered saline (PBS). Then, fresh MEM was added to the cells to continue cultivation. After 24 h of cell treatment, all liquid in each well was removed, and the cells were washed twice with PBS. Hoechst 33342 was used for nuclear staining. Finally, the cells were observed for the EGFP fluorescence (EX WL 488 nm, EM WL 597 nm) and photographed using an IXM-C high-intensity imaging analysis system.

To explore the optimal transfection time, the liposomes containing EGFP mRNA in the fixed condition was added to the culture Neuro-2a cells and incubated for 0, 3, 6, 12, 24, or 48 h. and then the cells was treated and evaluated by the EGFP fluorescence as described above.

### 2.4. Evaluation of the cytotoxicity in vitro

All the liposomes with the different cation:anion molar ratios 10:0, 7:3, 0:10 and content of lipid (4.8 mg/mL) were prepared as mentioned above. The prepared liposomes were diluted 10 times with MEM to obtain a solution with a concentration of 0.48 mg/mL. Next, the above solution was diluted to 1, 0.9, 0.8, 0.7, 0.6, 0.5, 0.4, 0.3, or 0.2 times with MEM. Then, these nine different concentrations of the liposome diluent were added to Neuro-2a cells. Each liposome diluent was added to six wells, with 150 µL per well. Subsequently, all the fluid in the wells was removed after the cells incubated for 24 h. MEM and CCK-8 reagent were mixed at a volume ratio of 10:1. Next, 110 µL of this mixture was added to each well and incubated for 1 h. The absorbance was measured at 450 nm.

### 2.5. Preparation of liposomes/LNP with different method

**Extrusion method:** DOTAP and POPS were weighed and mixed at a molar ratio of 7:3, and the subsequent sample treatment was consistent with the ultrasonic method mentioned above. The samples were extruded through a polycarbonate film with a pore size of 0.1 micron and repeated 10 times via a syringe extruder. Finally, liposome/mRNA complex was formed and collected.

**Mixing method**: DOTAP and POPS were weighed and mixed at a molar ratio of 7:3 and treated as mentioned above to form to film. And the only RNase-free water was added to the film respectively, and ultrasonicated for 5 min to prepare a blank liposome. Then, EGFP mRNA solution was added to the liposome and mixed for 10 min. Finally, mRNA was absorbed on the surface of blank liposomes.

**Microfluidic method for LNP**: DOTAP and POPS were weighed and mixed at a molar ratio of 7:3, dissolved in ethanol to obtain a 0.576 mg/mL solution. EGFP mRNA was dissolved in citric-acid–sodium-citrate buffer (pH = 4) to form a solution of 0.027 mg/mL, and the volume ratio of the ethanol phase to water phase was 1:3. The two phases were then mixed rapidly at a flow ratio of 1:3 through a microfluidic chip. The product was diluted immediately with PBS to a volume 30 times to decrease proportion of ethanol to stabilize the structure. Finally, the product was centrifugated in a 30KD ultrafiltration tube to remove ethanol.

The lipid nanoparticles (LNPs) loaded with luciferase mRNA were formed using this method (Cheng et al., 2020). The molar ratio of all lipids (DLin-MC3-DMA/DOTAP:POPS/DSPC:Cholesterol:DSPE-PEG2000) was 50.0:10.0:38.5:1.5. All lipids were mixed and dissolved in ethanol to obtain a 12 mM/L solution. Luciferase mRNA was dissolved in citric-acid–sodium-citrate buffer (pH = 4) to form a solution of 0.108 mg/mL, and the volume ratio of the ethanol phase to water phase was 1:3. The two phases were then mixed rapidly at a flow ratio of 1:3 through a microfluidic chip. The product was also diluted with PBS to a volume 30 times that of the ethanol to stabilize the structure. Finally, the product was centrifugated in a 30KD ultrafiltration tube to remove ethanol.

### 2.6. LNP transfection in vivo

BALB/C mice were 4 weeks old. The rodents had free access to sterilized food and distilled water and were maintained in stainless steel cages filled with hardwood chips in an air-conditioned room under 12:12 h light/dark cycles.

BALB/C mice were intravenously injected with LNPs. After 6 h of injection, the mice were intraperitoneally injected with d-Luciferin (15 mg/mL). Bioluminescence was imaged using the IVIS Spectrum imaging system under anesthesia of isoflurane mixed with oxygen.

### 2.7. Evaluation of LNP releasing

Each different type of LNP was placed at 37 °C for 0, 1, 3, 6, 12, 24, 36, or 48 h. Then, DAPI was mixed with LNPs or naked mRNA in a 3:1 volume ratio. The agarose was dissolved in 0.5% nucleic acid buffer solution TAE to form a solution with a mass fraction of 1%. 10 μL of Gel-Red were added to the solution per 100 mL and heated to melt. The agarose solution was poured into the mold, and a comb was inserted into it until solidification to create 15 wells. A mixture of DAPI and LNP or naked mRNA was added to the agarose gel at 20 μL per well. The gel was run at 120 V for 30 min.

## 3 Results and discussion

### 3.1. Design, synthesis, and characterization of liposome system

**Figure 1.**
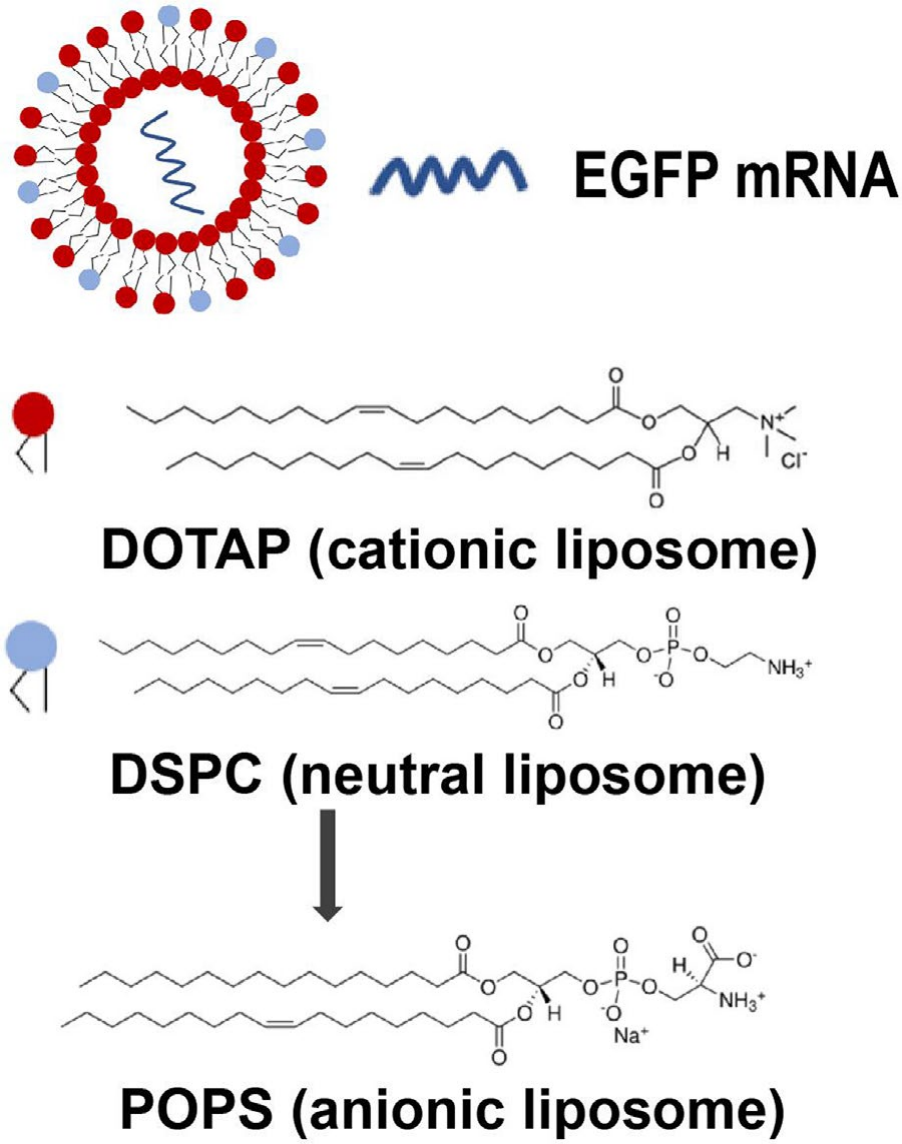
Lipid components of differently charged liposomes.

Traditional liposome (Figure 1) consists of cationic lipids, electrically neutral auxiliary phospholipids, cholesterol, and phospholipids containing polyethylene glycol (PEG) (Liu et al., 2021). The most classic and highly efficient cationic lipid, 1,2-Dioleoyl-3-trimethylammonium-propane (DOTAP), was chosen as the core cation component of the liposome to guarantee effective transfection (Simberg et al., 2004). In previous reports, electrically neutral 1,2-dioleoyl-sn-glycero-3-phosphoethanolamine (DOPE) or 1,2-distearoyl-sn-glycero-3-phosphocholine (DSPC) were applied as auxiliary phospholipids to form the skeleton of liposomes (Cheng & Lee, 2016; Yanez Arteta et al., 2018). However, in this study, phosphatidyl serine (PS) was applied to replace DOPE or DSPC for increasing the safety of the system because the PS as a negatively charged phospholipids can neutralize part of the positive charge of DOTAP (Zhao et al., 2022) and reduce the overall charge of the particle (Figure 1). EGFP mRNA was chosen as targeted mRNA loaded by liposome due to the accessibility of the fluorescence of EGFP expressed by the mRNA (Baeken & Yokobayashi, 2022; Larson et al., 2022).

### 3.2. In-vitro efficiency and safety of liposomes with different cation:anion molar ratios

**Figure 2.**
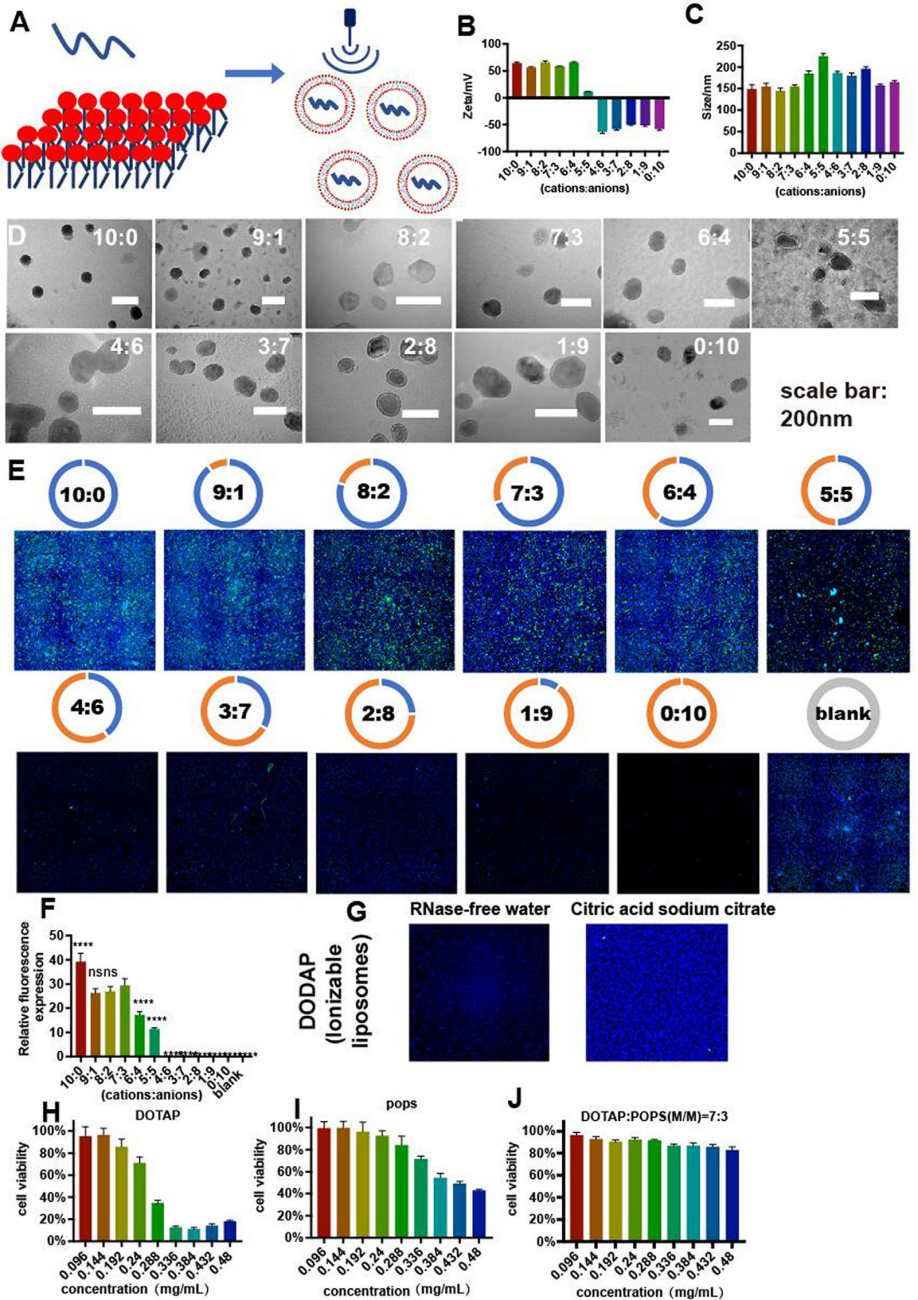
Influence of DOTAP and POPS with different molar ratios on transfection efficiency in vitro. **(A)** Preparation of liposome containing EGFP mRNA by ultrasonic dispersion method. **(B)** Zeta and **(C)** size of liposomes with different molar ratios. **(D)** Transmission electron microscopy of liposomes with different molar ratios of DOTAP and POPS (10:0, 9:1, 8:2, 7:3, 6:4, 5:5, 4:6, 3:7, 2:8, 1:9, 0:10). **(E)** EGFP (green) expression in murine Neuro-2a cells treated with liposomes containing EGFP mRNA and composed of different molar ratios of DOTAP and POPS. The total concentration of liposomes in all groups was 0.48 mg/mL, the concentration of EGFP mRNA was 0.027 mg/mL, and the nuclei (blue) were stained with Hoechst 33342. **(F)** Relative fluorescence expression (fluorescence intensity/hole area) of the cells in each group. *p** **<** **0.05, **p** **<** **0.01, ***p** **<** **0.001, ****p** **<** **0.0001 (*n* = 3). **(G)** EGFP (green) expression treated with liposome composed of DODAP. **(H–J)** Cell viability of Neuro-2a cells after treatment with different concentrations of DOTAP, POPS, and liposome of DOTAP/POPS (M/M) = 7:3 for 24 h.

A series of liposomes with different cation:anion molar ratios of lipids were synthesized by ultrasonic dispersion method (Figure 2(A)). With a decrease in the proportion of cationic lipids from 100% to 0%, the surface potential and size of the liposome gradually changed from about 50 to −50 mV (Figure 2(B)) and 148.6 to 225.1 nm, respectively (Figure 2(C)). However, the spherical liposome morphology remained the same when the surface potential was changed, as evaluated by transmission electron microscopy (TEM; Figure 2(D)). When the cation:anion molar ratio was 5:5, the liposome had the largest size and nearly zero total surface charge, indicating that the overall charge of the liposome had a crucial influence on the stability of the system, and agglomeration and precipitation were prone to occur under low-charge conditions.

The transfection effect of liposomes with different cation:anion molar ratios was evaluated in vitro. The liposome composed of 100% DOTAP showed the highest transfection efficiency. When the ratio of DOTAP was 70–90%, there was no significant difference in the EGFP mRNA transfection efficiency (Figure 2(E)). As the proportion of DOTAP continued to decline, no fluorescence of the EGFP was observed when it dropped to 50% (Figure 2(F)). When the cationic lipids DOTAP was replaced by the ionizable lipids DODAP in the same ratio (7:3 M/M), the new liposome showed no transfection effect in either RNase-free water or acidic liquid (Figure 2(G)). Therefore, considering the balance between transfection efficiency and cytotoxicity of the positive charge lipids, the liposomes with as little DOTAP proportion as possible were chosen (cation:anion molar ratio of 7:3) as our optimal delivery system.

The cytotoxicity of the liposome with different cation:anion molar ratios was evaluated by CCK-8 method (Li et al., 2022b). The cationic lipids DOTAP caused nearly 65% cell apoptosis when the concentration reached 0.288 mg/mL (Figure 2(H)). The anionic lipids POPS showed no obvious cytotoxicity (Figure 2(I)) at the concentration of 0.288 mg/mL, but nearly 50% cell apoptosis at high concentration of 0.48 mg/mL. However, when DOTAP was mixed with POPS at the molar ratio of 7:3 as mentioned above, cell viability was above 80%, even at a concentration of 0.48 mg/mL, which was much safer than each individual component (Figure 2(J)). In summary, the appropriate addition of anionic lipids would increase the safety of the entire liposome system, while maintaining the transfection efficiency.

### 3.3. Transfection evaluation with structures of different binding modes between mRNA and liposome

**Figure 3.**
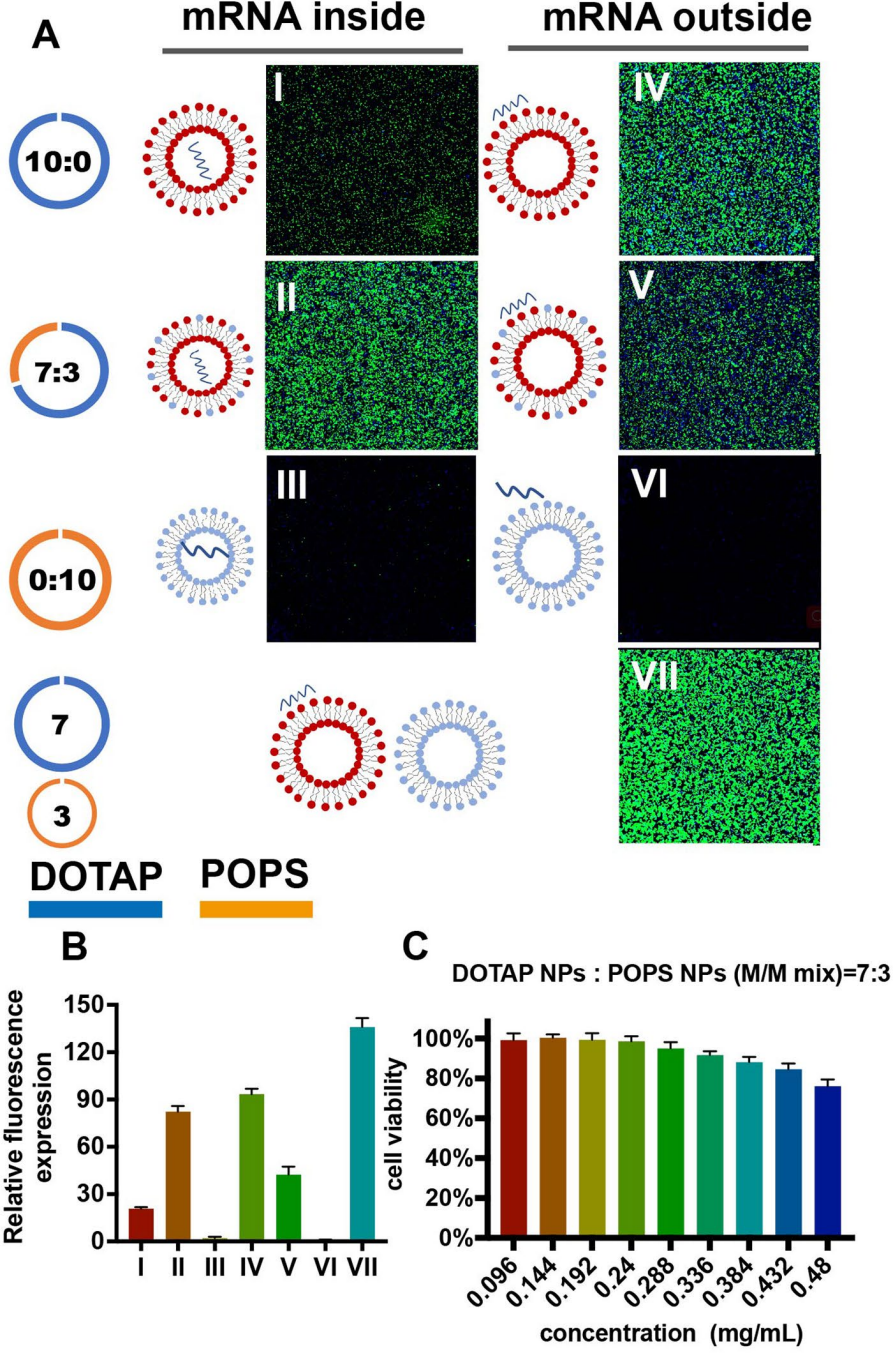
**(A)** EGFP (green) expression in murine Neuro-2a cells treated with liposomes containing EGFP mRNA with different structures. **(B)** Relative fluorescence expression (fluorescence Intensity/hole area) of the cells in each group (*n* = 3). **(C)** Cell viability of Neuro-2a cells after treatment with liposomes with different structures.

The transfection effects of liposome combining with mRNA in different structures were investigated. Blank liposomes without loaded mRNA were prepared in the same ratio. Effective in-vitro transfection can also be obtained when blank liposomes mixed with mRNA solution, which meant mRNA can be effectively transfected not only by being encapsulated in the liposomes (Figure 3(A) II), but also by being adsorbed on the surface of the particles (Figure 3(A) V). Moreover, each component of the lipids was prepared to nanoparticles, respectively, with different mRNA combining structures (encapsulating (Figure 3(A) I, III) or adsorbing (Figure 3(A) IV, VI). Only cation-based liposomes could achieve effective transfection of mRNA into cells, regardless of the mRNA structure applied to the liposome (Figure 3(B)). Furthermore, when the DOTAP nanoparticles, POPS nanoparticles and mRNA solution were mixed to a suspension at the molar ratio of 7:3 (Figure 3(A) VII), mRNA could still be effectively transfected, which meant that the suspension could still work even if the positively and negatively charged materials were not in a liposome unit. The mixed liposome suspension showed no obvious cytotoxicity (Figure 3(C)), as did previously tested ones (Figure 2(J)). Together, these data revealed that phospholipids and mRNA composed in all structures achieved effective transfection, showing that the charge and other biochemical properties of the phospholipids played a more decisive role in transfection than the physical structure.

### 3.4. Transfection effect of charge in nanoparticles

**Figure 4.**
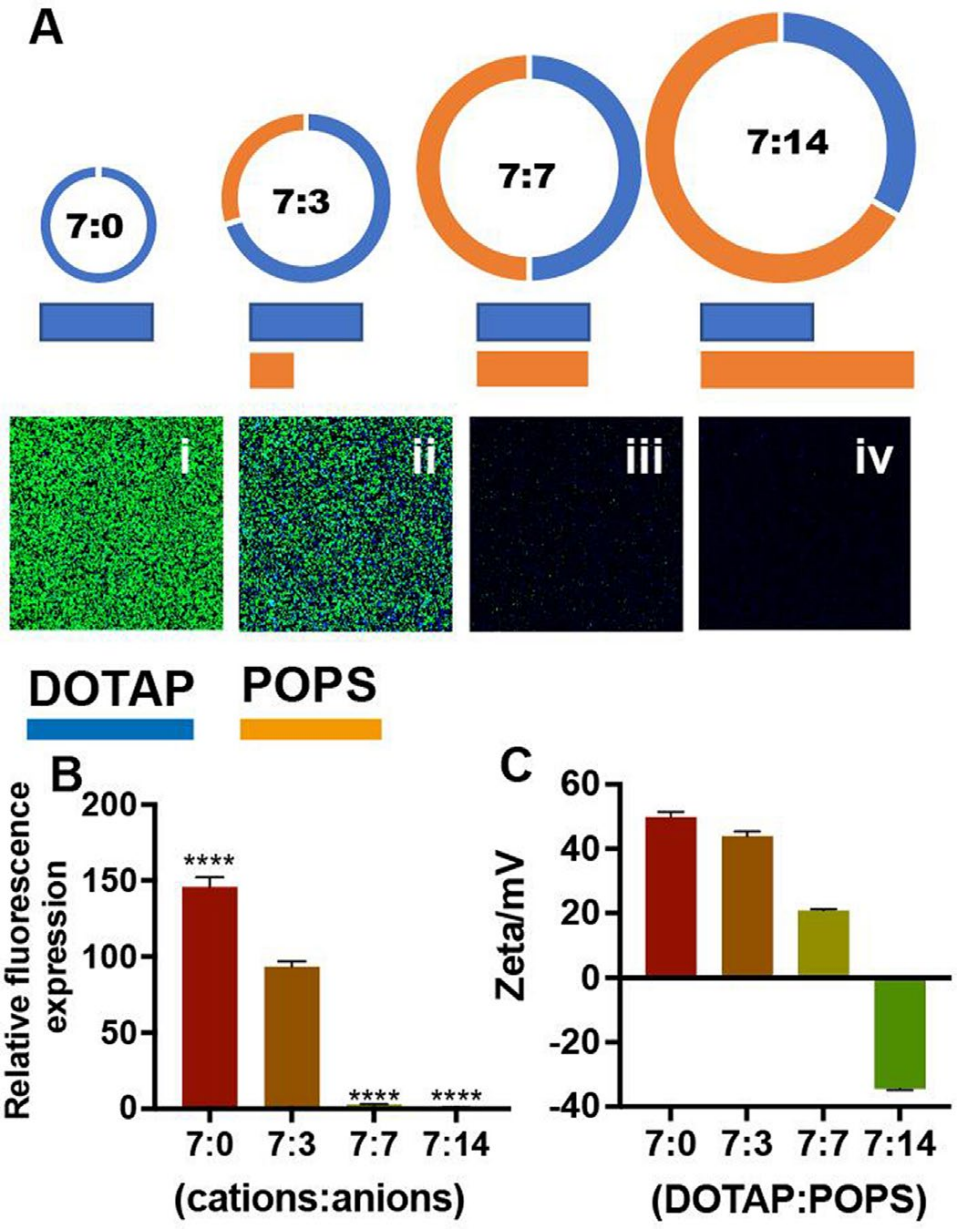
**(A)** EGFP expression in Neuro-2a cells treated with liposomes containing EGFP mRNA with different molar ratios of DOTAP and POPS (7:0, 7:3, 7:7, and 7:14) for 24 h. The amount of DOTAP was the same in each group, the concentration of EGFP mRNA was 0.027 mg/mL, the concentration of total liposomes in the 7:3 group was 0.192 mg/mL, and the nuclei (blue) were stained with Hoechst 33342. **(B)** Relative fluorescence expression (fluorescence intensity/hole area) of the cells in each group. *p** **<** **0.05, **p** **<** **0.01, ***p** **<** **0.001, ****p** **<** **0.0001 (*n* = 3). **(C)** Zeta of liposomes with different molar ratios.

Further clarification was needed regarding whether the absolute amount or the proportion of positive charges played a more important role in transfection efficiency. A series of liposomes composed of cationic lipids (DOTAP) with a fixed mass and anionic lipids (POPS) of different masses were designed and synthesized by mixing method. After transfection of cells for 24 h, fluorescent images of EGFP showed that liposome (DOTAP 0.1328 mg/mL, POPS 0 mg/mL, 7:0 molar ratio) could effectively transfect mRNA into proteins (Figure 4(A) i, (B)). When the cationic lipids content in the system remained unchanged, the liposome transfection efficiency gradually decreased with an increase amount of anionic lipids (Figure 4(A) ii, DOTAP 0.1328 mg/mL, POPS 0.0638 mg/mL, 7:3 molar ratio). The liposomes completely lost the ability to transfect mRNA when the increased anionic lipids shielded most of the positively charged material (7:7 and 7:14 molar ratio, Figure 4(A) iii, iv and (C)). These results suggested that the key point of transfection efficiency of the liposome was the proportion of the core cationic lipids in the whole liposome system, but not its dosage.

### 3.5. Transfection effects of cholesterol and total concentration

**Figure 5.**
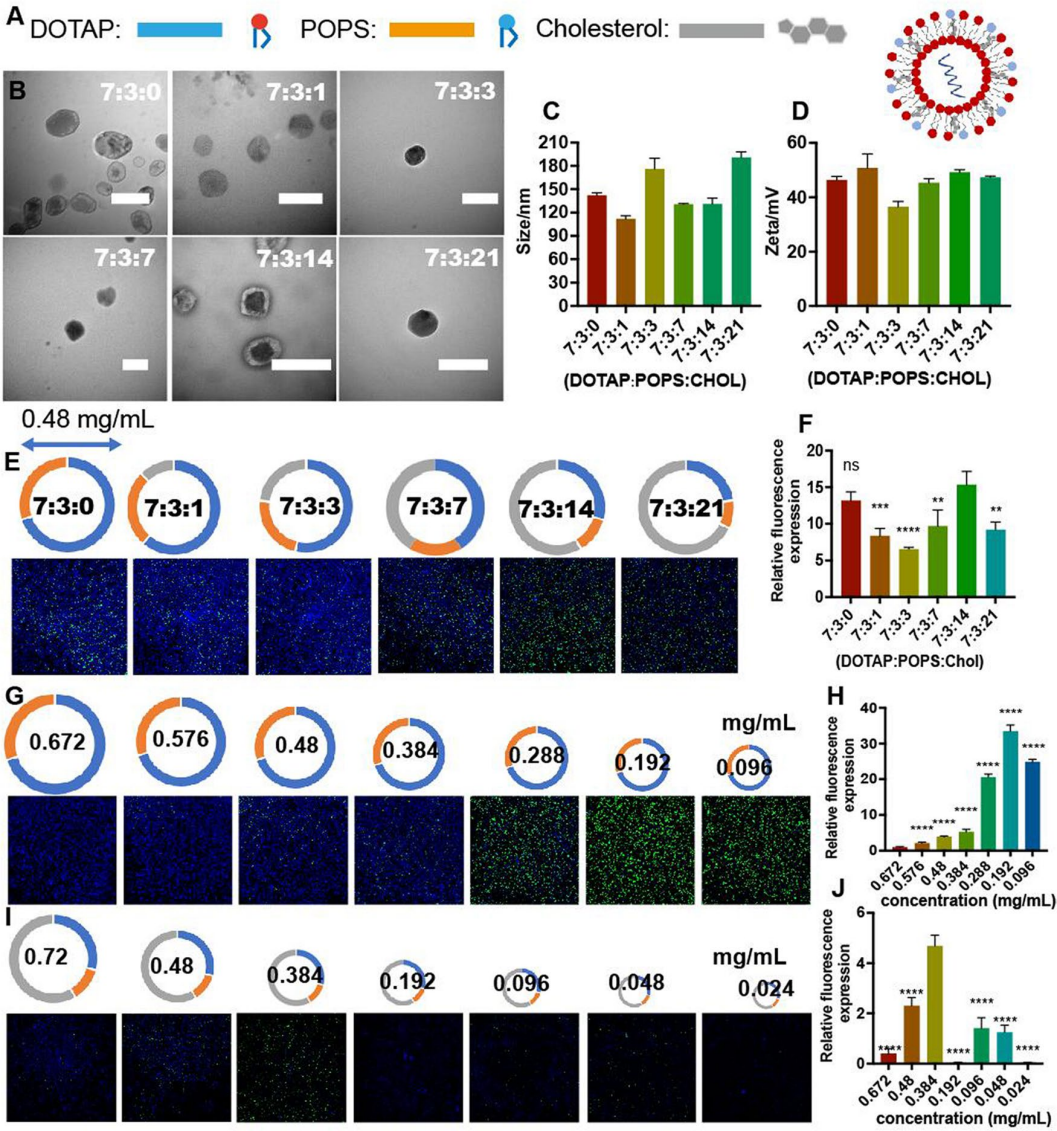
**(A)** Illustration of cholesterol-doped liposomes. **(B)** Transmission electron microscopy images of liposomes containing EGFP mRNA and composed of different molar ratios of DOTAP, POPS, and cholesterol (7:3:0, 7:3:1, 7:3:3, 7:3:7, 7:3:14, and 7:3:21). **(C)** Size and **(D)** Zeta of liposomes with different molar ratios. **(E)** EGFP expression in murine Neuro-2a cells treated with liposomes containing EGFP mRNA and composed of different molar ratios of lipids after 24 h. The total concentration of liposomes in all groups was 0.48 mg/mL, the concentration of EGFP mRNA was 0.027 mg/mL, and the nuclei (blue) were stained with Hoechst 33342. **(F)** Relative fluorescence expression (fluorescence intensity/hole area) of the cells in each group. **(G)** EGFP expression in Neuro-2a cells treated with different total concentration of liposomes containing EGFP mRNA after 24 h. DOTAP/POPS (M/M) = 7:3, the concentration of EGFP mRNA was 0.027 mg/mL, and the nuclei (blue) were stained with Hoechst 33342. **(H)** Relative fluorescence expression (fluorescence intensity/hole area) of the cells in each group. **(I)** EGFP (green) expression in Neuro-2a cells treated with different total concentration of liposomes containing EGFP mRNA after 24 h. DOTAP/POPS/cholesterol (M/M/M) = 7:3:14, the concentration of EGFP mRNA was 0.027 mg/mL, and the nuclei (blue) were stained with Hoechst 33342. **(J)** Relative fluorescence expression (fluorescence intensity/hole area) of the cells in each group. *p** **<** **0.05, **p** **<** **0.01, ***p** **<** **0.001, ****p** **<** **0.0001 (*n* = 3).

Cholesterol was another basic auxiliary material of liposomes (Ruwizhi & Aderibigbe, 2020), which has no charge but a tiny structure, can fill the gaps in the liposome membrane to stabilize the membrane structure (Clarke, 2019; Luo et al., 2020). Based on the optimal molar ratio (7:3) of DOTAP:POPS determined above, the influence of cholesterol on the liposomes was further investigated (Figure 5(A)). Different proportions of cholesterol were added to the system with different proportions form 7:3:0 to 7:3:21 (DATAP:POPS:Chol, molar ratio) and synthesized using the same method. All cholesterol-doped liposomes showed similar spherical shapes (Figure 5(B)) approximately 200 nm in size (Figure 5(C)) and with a surface potential change of 50 mV (Figure 5(D)). The addition of cholesterol stabilized the liposome’s structure and achieved an optimal transfection effect when the molar ratio of cholesterol was approximately 58% (DOTAP:POPS:cholesterol = 7:3:14). However, a further increase in the cholesterol ratio decreased the transfection effect due to the dilution of functional lipid components (Figure 5(E) and (F)). As long as the proportion of functional lipids (DOTAP and POPS) exceeded 42%, the whole liposome would take effect, which means that 58% of the liposome space could be used for subsequent addition of other functional components.

The appropriate ratio of lipids to mRNA was also a key factor for transfection effect. In the groups of liposomes without cholesterol, EGFP mRNA was transfected effectively when the total concentration of lipids (DOTAP:POPS = 7:3) was lower than 0.48 mg/mL. The best effect was achieved when the total concentration was 0.192 mg/mL, which was the ratio of 7.1:1 (lipids to mRNA (W/W)) (Figure 5(G) and (H)). In the liposomes with the optimal ratio of DOTAP:POPS:cholesterol (7:3:14), the best effect was achieved when the concentration of lipids was 0.384 mg/mL (m_lipids_:m_mRNA_=14.2:1) (Figure 5(I) and (J)). More specifically, the actual effective concentration of functional lipid components was 0.184 mg/mL in the liposome (DOTAP:POPS:cholesterol = 7:3:14, M/M/M), which was close to the data from the non-cholesterol liposome group (0.192 mg/mL). This reflected that the main functional liposome components (DOTAP and POPS) were the core influencing factors in liposomes.

### 3.6. Transfection evaluation of onset time

**Figure 6.**
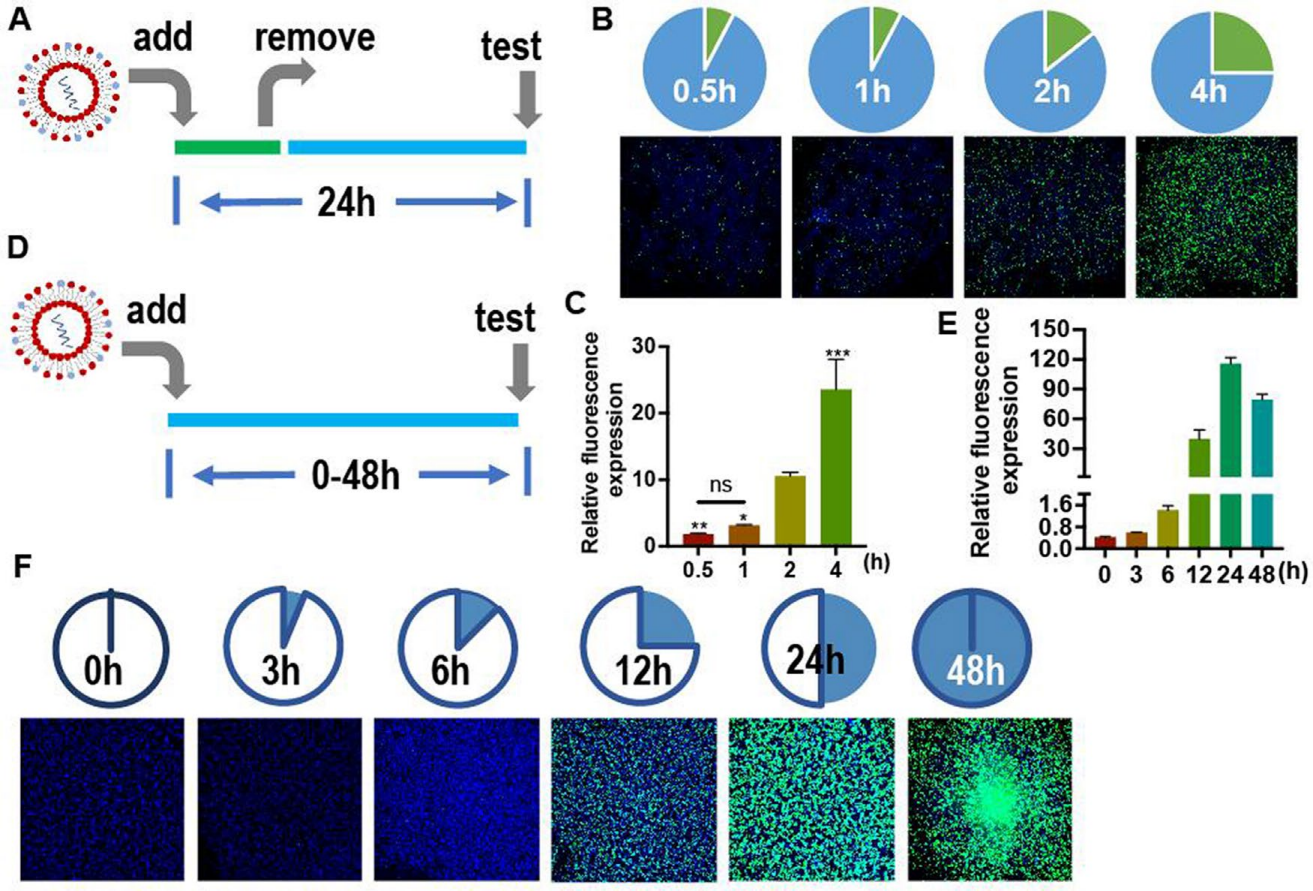
**(A)** Illustration of EGFP (green) expression with different liposome uptake times (0.5, 1, 2, or 4 h). **(B)** EGFP (green) expression in murine Neuro-2a cells treated with different uptake times. **(C)** Relative fluorescence expression (fluorescence intensity/hole area) of the cells in each group. **(D)** Illustration of EGFP (green) expression after different transfection times (0, 3, 6, 12, 24, or 48 h). **(E)** Relative fluorescence expression (fluorescence intensity/hole area) of the cells in each group. **(F)** EGFP (green) expression in Neuro-2a cells over time.

Since the transfection process included two steps, cell entry and protein expression, the onset time required for each stage was evaluated by monitoring cell fluorescence. Cells were incubated with liposomes for a certain period of time (0.5, 1, 2, or 4 h), and then the final fluorescence expression was measured after 24 h (Figure 6(A)). The results showed that only a small amount of liposomes were absorbed by cells within 2 h. It took at least 4 h for the liposome–mRNA complexes to effectively and sufficiently enter cells for subsequent effective transfection (Figure 6(B) and (C)). In the next stage of protein expression, cellular fluorescence was continuously monitored during a period of time (0, 3, 6, 12, 24, or 48 h) (Figure 6(D)). Almost no fluorescence was detected before 6 hours of mRNA expression. EGFP mRNA needed at least 12 h to achieve effective transfection, expressed the best effect after 24 h, and continued working for 48 h (Figure 6(E) and (F)). In summary, the liposome containing EGFP mRNA required 4 h to enter cells sufficiently, needed at least 8 h to take effect and fully express after 20 h.

### 3.7. Transfection evaluation of the preparation methods

**Figure 7.**
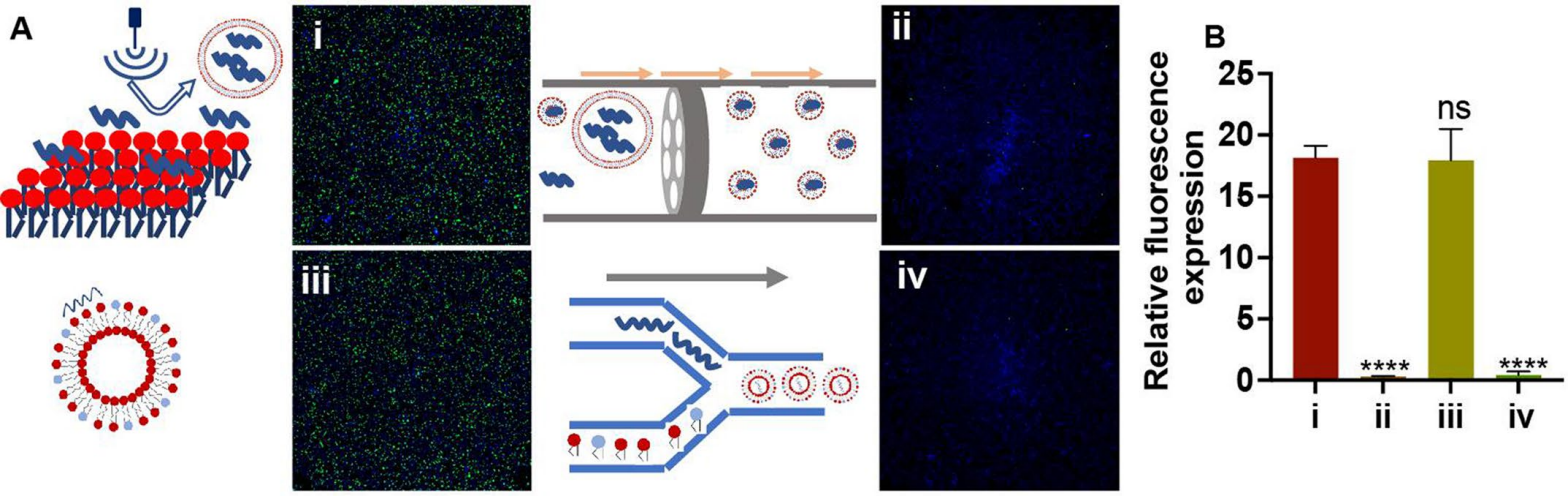
**(A)** Structure and EGFP (green) expression in murine Neuro-2a cells treated with liposomes containing EGFP mRNA that were prepared via different methods (i. ultrasonic dispersion method, ii. extrusion method, iii. mixing method, or iv. microfluidic method). **(B)** Relative fluorescence expression (fluorescence intensity/hole area) of the cells in each group. *p** **<** **0.05, **p** **<** **0.01, ***p** **<** **0.001, ****p** **<** **0.0001 (*n* = 3).

Liposomes were prepared using other classic methods, such as the extrusion method (Figure 7(A) ii), mixing method (Figure 7(A) iii), and microfluidic method (Figure 7(A) iv) in the same cation:anion molar ratios and concentrations of best efficacy as described above (Figure 5(I)). The results showed that liposomes prepared by simple physical fusion and dispersion methods, such as the ultrasonic dispersion method (Figure 7(A) i) and mixing method, could achieve effective transfection. However, samples prepared by the extrusion method were ineffective, which was probably caused by the mRNA decomposed or adsorbed with the filter membrane during the repeated extrusion process. Therefore, the process of liposome preparation should avoid excessive addition of other materials and should be as simple as possible so as to not damage the fragile mRNA (Figure 7(B)).

It is worth noting that the LNPs prepared via the popular microfluidic method (Figure 7(A) iv) using the same materials and cation:anion molar ratios did not show significant transfection, which might be caused by residual ethanol (Figure 7(B)) or too tight binding force between DOTAP and POPS. These results showed that different preparation methods had an important influence on the final effect of liposomes.

### 3.8. Evaluation of the delivery system in vivo

**Figure 8.**
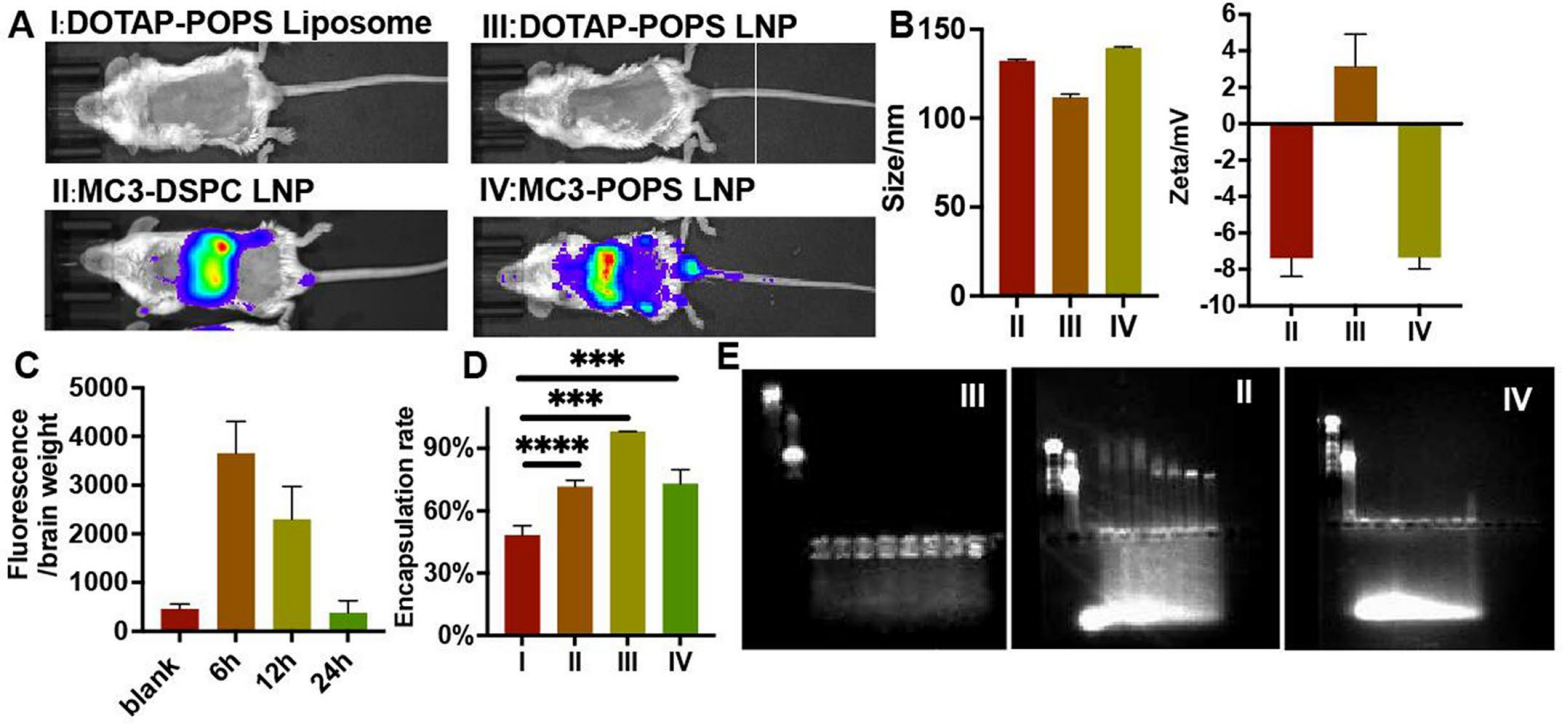
**(A)** The live luciferase fluorescence imaging of mice treated with different mRNA liposomes. **(B)** The size (left) and Zeta potential (right) of different liposomes, *n* = 3. **(C)** mRNA transfected in mice at different times. **(D)** the encapsulation rate of liposome. **(E)** Gel electrophoresis of different mRNA liposome. Samples in each gel are DNA marker, mRNA solution, LNPs releasing in 48h, 36h, 24h, 12h, 6h, 3h, 1h, 0h, respectively from right to left.

Liposome-mediated mRNA transfection in vivo was finally evaluated. Unfortunately, the liposome of DOTAP with POPS (type I), which was highly expressed in vitro, was not expressed at the animal level (Figure 8(A) I). In comparison, the LNP composed of MC3 and DSPC prepared via the microfluidic method (type II) could effectively transfect luciferase mRNA in mice (Figure 8(A) II), but the LNP composed of DOTAP and POPS prepared via the same method (type III) showed no transfection effect (Figure 8(A) III). This result preliminarily showed that the synthesis method was not the only factor for mRNA transfection in vivo. After replacing the neutral lipid DSPC with anionic POPS (type IV) in the LNP, effective in-vivo transfection was also achieved after 6 h (Figure 8(C)), showing that the material difference between MC3 and DOTAP was the core factor influencing the expression of the mRNA delivery system in vivo.

The properties of all nanoparticles were further evaluated to explore the transfection factors in vivo. The size of all the added LNPs was below 150 nm (Figure 8(B)), and the surface charge of nanoparticles with DOTAP as the key component was positive, and the rest were negative (Figures 8(B) and (C)). Liposomes prepared via the ultrasonic dispersion method (type I) had a low encapsulation rate (Figure 8(D)), which meant that a large amount of mRNA could not be effectively loaded and protected by the liposome, resulting in no expression in mice. Type III LNPs, with the same components as type I, were synthesized via the microfluidic method, showing high encapsulation rate; however, type-III LNPs still not transfect mRNA in mice owing to the lack of mRNA release from the LNPs even after 48 h due to strong binding of DOTAP with mRNA (Figure 8(E)). In summary, for effective mRNA expression in vivo, the complex and strict requirements about sufficient encapsulation and rapid drug releasing for the mRNA delivery system were necessary, while were not needed in vitro.

## 4. Conclusions

The effects of liposome composition on transfection in detail were investigated. In brief, we demonstrated that adding an appropriate amount of the anionic lipid POPS to liposomes reduced the strong positive charge of the liposomes, thereby increasing the safety of the delivery system while maintaining the original transfection efficiency. The liposome composed of DOTAP and POPS at a molar ratio of 7:3 achieved the best balance between safety and efficacy. When the liposomes were evaluated in vitro, the proportion of positively charged cationic lipids in the liposome determined the efficiency of the whole system, rather than the structure of the mRNA loading form. The liposome can achieve mRNA transfection only if the proportion of positively charged materials take the major composition in the system. There was optimal usage (7.1:1, lipids to mRNA (W/W)) for the overall concentration of liposomes. The liposome system required at least 4 h to completely enter the cells and reach its transfection peak at 24 h. Even with optimal lipid composition and proportions, the liposome synthesis method had a crucial influence on transfection efficiency. If mRNA system only worked in vitro or in screening stage, the ultrasonic method was the best appropriate choice due to the advantages of simple, cost-effective, and lower sample consumption. If considering the mRNA expression at the animal level and scalable production, microfluidic method was the best technology to achieve suitable mRNA encapsulation and releasing rate and batch production with stable quality control. Based on this, by accurately adjusting the final overall charges, more liposomes with cationic lipids for high transfection efficiency and safety can be designed and synthesized, providing another approach aside from ionizable lipids to build liposome systems. For transporting mRNA in vivo, requirements regarding the mRNA encapsulation and releasing rate should be further considered to optimize liposome design and preparation.

## Data Availability

The datasets used or analyzed during the current study are available from the corresponding author on reasonable request.

## References

[CIT0001] Anthony K. (2022). RNA-based therapeutics for neurological diseases. RNA Biol 19:1–11. 35067193 10.1080/15476286.2021.2021650 PMC8786337

[CIT0002] Baeken MW, Yokobayashi Y. (2022). Identification of an ERN1 target site within EGFP mRNA. J Cell Biochem 123:1298–305. 35908204 10.1002/jcb.30314 PMC9544080

[CIT0003] Bogaert B, Sauvage F, Guagliardo R, et al. (2022). A lipid nanoparticle platform for mRNA delivery through repurposing of cationic amphiphilic drugs. J Control Release 350:256–70. 35963467 10.1016/j.jconrel.2022.08.009 PMC9401634

[CIT0004] Carrasco MJ, Alishetty S, Alameh MG, et al. (2021). Ionization and structural properties of mRNA lipid nanoparticles influence expression in intramuscular and intravascular administration. Commun Biol 4:956. 34381159 10.1038/s42003-021-02441-2 PMC8358000

[CIT0005] Cheng X, Lee RJ. (2016). The role of helper lipids in lipid nanoparticles (LNPs) designed for oligonucleotide delivery. Adv Drug Deliv Rev 99:129–37. 26900977 10.1016/j.addr.2016.01.022

[CIT0006] Cheng Q, Wei T, Farbiak L, et al. (2020). Selective organ targeting (SORT) nanoparticles for tissue-specific mRNA delivery and CRISPR-Cas gene editing. Nat Nanotechnol 15:313–20. 32251383 10.1038/s41565-020-0669-6 PMC7735425

[CIT0007] Clarke RJ. (2019). Effect of cholesterol on the dipole potential of lipid membranes. Adv Exp Med Biol 1115:135–54. 30649758 10.1007/978-3-030-04278-3_6

[CIT0008] Durymanov M, Reineke J. (2018). Non-viral delivery of nucleic acids: insight into mechanisms of overcoming intracellular barriers. Front Pharmacol 9:971. 30186185 10.3389/fphar.2018.00971 PMC6111240

[CIT0009] Eygeris Y, Gupta M, Kim J, Sahay G. (2022). Chemistry of lipid nanoparticles for RNA delivery. Acc Chem Res 55:2–12. 34850635 10.1021/acs.accounts.1c00544

[CIT0010] Forster Iii J, Nandi D, Kulkarni A. (2022). mRNA-carrying lipid nanoparticles that induce lysosomal rupture activate NLRP3 inflammasome and reduce mRNA transfection efficiency. Biomater Sci 10:5566–82. 35971974 10.1039/d2bm00883a

[CIT0011] Koynova R, Tenchov B. (2011). Recent patents in cationic lipid carriers for delivery of nucleic acids. Recent Pat DNA Gene Seq 5:8–27. 21288191 10.2174/187221511794839255

[CIT0012] Larson NR, Hu G, Wei Y, et al. (2022). pH-dependent phase behavior and stability of cationic lipid-mRNA nanoparticles. J Pharm Sci 111:690–8. 34774918 10.1016/j.xphs.2021.11.004

[CIT0013] Leventis PA, Grinstein S. (2010). The distribution and function of phosphatidylserine in cellular membranes. Annu Rev Biophys 39:407–27. 20192774 10.1146/annurev.biophys.093008.131234

[CIT0014] Li M, Li Y, Li S, et al. (2022a). The nano delivery systems and applications of mRNA. Eur J Med Chem 227:113910. 34689071 10.1016/j.ejmech.2021.113910 PMC8497955

[CIT0015] Li N, Sun Y, Fu Y, Sun K. (2021). RNA drug delivery using biogenic nanovehicles for cancer therapy. Front Pharmacol 12:734443. 35002692 10.3389/fphar.2021.734443 PMC8740118

[CIT0016] Liu S, Cheng Q, Wei T, et al. (2021). Membrane-destabilizing ionizable phospholipids for organ-selective mRNA delivery and CRISPR-Cas gene editing. Nat Mater 20:701–10. 33542471 10.1038/s41563-020-00886-0 PMC8188687

[CIT0017] Li Y, Zhuang Q, Tao L, et al. (2022b). Urolithin B suppressed osteoclast activation and reduced bone loss of osteoporosis via inhibiting ERK/NF-κB pathway. Cell Prolif 55:e13291. 35708050 10.1111/cpr.13291 PMC9528769

[CIT0018] Luo J, Yang H, Song BL. (2020). Mechanisms and regulation of cholesterol homeostasis. Nat Rev Mol Cell Biol 21:225–45. 31848472 10.1038/s41580-019-0190-7

[CIT0019] Lv H, Zhang S, Wang B, et al. (2006). Toxicity of cationic lipids and cationic polymers in gene delivery. J Control Release 114:100–9. 16831482 10.1016/j.jconrel.2006.04.014

[CIT0020] Ramachandran S, Satapathy SR, Dutta T. (2022). Delivery strategies for mRNA vaccines. Pharmaceut Med 36:11–20. 35094366 10.1007/s40290-021-00417-5 PMC8801198

[CIT0021] Ruwizhi N, Aderibigbe BA. (2020). The efficacy of cholesterol-based carriers in drug delivery. Molecules 25:4330. 32971733 10.3390/molecules25184330 PMC7570546

[CIT0022] Schlake T, Thess A, Fotin-Mleczek M, Kallen KJ. (2012). Developing mRNA-vaccine technologies. RNA Biol 9:1319–30. 23064118 10.4161/rna.22269 PMC3597572

[CIT0023] Seki H. (2018). Complications with vacuum delivery from a forceps-delivery perspective: Progress toward safe vacuum delivery. J Obstet Gynaecol Res 44:1347–54. 29974574 10.1111/jog.13685

[CIT0024] Simberg D, Weisman S, Talmon Y, Barenholz Y. (2004). DOTAP (and other cationic lipids): chemistry, biophysics, and transfection. Crit Rev Ther Drug Carrier Syst 21:257–317. 15638468 10.1615/critrevtherdrugcarriersyst.v21.i4.10

[CIT0025] Tenchov R, Bird R, Curtze AE, Zhou Q. (2021). Lipid nanoparticles─from liposomes to mrna vaccine delivery, a landscape of research diversity and advancement. ACS Nano 15:16982–7015. 34181394 10.1021/acsnano.1c04996

[CIT0026] To KKW, Cho WCS. (2021). An overview of rational design of mRNA-based therapeutics and vaccines. Expert Opin Drug Discov 16:1307–17. 34058918 10.1080/17460441.2021.1935859

[CIT0027] Webb C, Ip S, Bathula NV, et al. (2022). Current status and future perspectives on mRNA drug manufacturing. Mol Pharm 19:1047–58. 35238565 10.1021/acs.molpharmaceut.2c00010

[CIT0028] Yanez Arteta M, Kjellman T, Bartesaghi S, et al. (2018). Successful reprogramming of cellular protein production through mRNA delivered by functionalized lipid nanoparticles. Proc Natl Acad Sci U S A 115:E3351–E3360. 29588418 10.1073/pnas.1720542115 PMC5899464

[CIT0029] Zhang S, Xu Y, Wang B, et al. (2004). Cationic compounds used in lipoplexes and polyplexes for gene delivery. J Control Release 100:165–80. 15544865 10.1016/j.jconrel.2004.08.019

[CIT0030] Zhao X, Ma X, Dupius JH, et al. (2022). Negatively charged phospholipids accelerate the membrane fusion activity of the plant-specific insert domain of an aspartic protease. J Biol Chem 298:101430. 34801553 10.1016/j.jbc.2021.101430 PMC8683733

